# Individual Differences in Testosterone and Self-Control Predict Compulsive Sexual Behavior Proneness in Young Males

**DOI:** 10.3389/fpsyg.2021.723449

**Published:** 2021-12-03

**Authors:** Geraldine Rodríguez-Nieto, Marieke Dewitte, Alexander T. Sack, Teresa Schuhmann

**Affiliations:** ^1^Department of Cognitive Neuroscience, Faculty of Psychology and Neuroscience, Maastricht University, Maastricht, Netherlands; ^2^Movement Control and Neuroplasticity Research Group, Group Biomedical Sciences, Department of Movement Sciences, KU Leuven, Heverlee, Belgium; ^3^Department of Clinical Psychological Science, Faculty of Psychology and Neuroscience, Maastricht University, Maastricht, Netherlands

**Keywords:** sexual compulsivity, testosterone, self-control, compulsive sexual behavior, sexual excitation, sexual inhibition, intercourse, masturbation

## Abstract

The ability to control sexual urges and impulses is essential to achieve individual and social harmony. Failing to regulate sexual behavior can lead to the interference with daily life goals, sexual diseases transmission and moral violations, among others. The dual control model of sexual response proposes that an imbalance between sexual excitation and sexual inhibition mechanisms may lead to sexual dysregulation, thereby explaining problematic sexual behavior. Interindividual differences in self-control and testosterone levels are likely to modulate sexual regulation mechanisms, but these individual features have scarcely been studied in the context of compulsive sexual behavior. This study investigated the role of sexual excitation and inhibition, self-control and testosterone levels in predicting individuals’ proneness to display compulsive sexual behavior. Seventy healthy young males provided a saliva sample for testosterone measurements and filled in questionnaires on self-control, sexual excitation, sexual inhibition, sexual compulsivity and sexual behavior. High testosterone levels and low self-control were associated with higher sexual compulsivity scores. Additionally, testosterone levels and sexual inhibition negatively predicted the frequency of sexual behavior with a partner. The results of our study highlight the joint role of psychological traits and testosterone levels in compulsive sexual behavior proneness, providing implications regarding the prevention and treatment of this condition in young males.

## Introduction

The ability to control sexual impulses and urges is essential to preserve individual health and harmonious social relationships. An excessive sexual preoccupation and a lack of control over sexual behavior can lead to a wide range of undesired consequences such as the disruption of daily goals, sexual disease transmission, undesired pregnancy and social norm violations. Whereas the judgment of the impact of excessive sexual behavior can be subject to social and cultural norms, uncontrolled sexual behavior is characterized by the negative impact on the life of the individual. In particular, compulsive sexual behavior disorder is defined as a persistent inability to control intense, repetitive sexual impulses or urges, affecting familial, social, educational, and/or occupational areas of functioning (World Health Organization, 2020). The clinical validity and legal implications of this condition have been extensively debated, leading to its inclusion as an impulse control disorder by the International Classification of Diseases, Eleven Revision (World Health Organization, 2020), but to the rejection of a similar clinical model (hypersexuality) by the DSM-IV (American Psychiatric Association, 2013).

Symptoms can emerge in different patterns and at different levels of intensity, thereby not always reaching clinical levels (Kafka, 2010). This implies that some individuals exhibit sexual compulsivity tendencies that have an impact on daily life, without being diagnosed. In college students, for example, higher sexual compulsivity scores have been associated with an increased frequency of sexual risk behaviors and perceived likelihood of negative outcomes such as distress or undesired pregnancy (Dodge et al., 2004; McBride et al., 2008). Therefore, identifying the predictors of compulsive sexual behavior proneness in non-clinical samples is relevant to reduce the probability of risky sexual behaviors and in preventing the development of pathological conditions.

Different individual, social and biological factors may underlie the lack of control over sexual behavior. Regarding the individual factors, the dual control model of sexual response proposes that individuals vary in their propensity to be sexually excited vs. sexually inhibited and is the balance that leads to a successful sexual regulation (Bancroft and Janssen, 2000). In this model, sexual excitation refers to the processes that increase the likelihood of sexual arousal, whereas sexual inhibition refers to processes that decrease the likelihood of its occurrence. Based on self-reports (Sexual Inhibition Scale—SIS) Bancroft and Janssen identified two types of sexual inhibition: (1) Sexual Inhibition (SIS1) caused by a threat of sexual performance failure which likely comprises peripheral mechanisms; and (2) Sexual Inhibition (SIS2) caused by the threat of performance consequences (e.g., risk of getting a contagious disease).

The dual control model postulates that individuals who are more easily sexually aroused (i.e., score higher in sexual excitation) and are low in the second type of sexual inhibition (SIS2—sexual inhibition due to negative consequences of sexual activity), are more prone to develop sexual compulsivity (Bancroft et al., 2009). Indeed, some studies have consistently found that higher scores on the Sexual Excitation Scale (SES) are associated with compulsive (or hypersexual) sexual behavior (Bancroft and Vukadinovic, 2004; Janssen and Bancroft, 2007; Winters et al., 2010; Pachankis et al., 2014; Rettenberger et al., 2016; Efrati and Mikulincer, 2018). However, the relationship between sexual compulsivity (or hypersexuality) and low sexual inhibition (SIS2) is less consistent, ranging from non-existing to modest (Bancroft and Vukadinovic, 2004; Pachankis et al., 2014; Miner et al., 2016; Rettenberger et al., 2016; Walton et al., 2017; Efrati and Mikulincer, 2018). This inconsistency could be explained by the fact that sexual inhibition (SIS2) is low specifically in self-defined *sex addicts* whose main way of acting out is not masturbation, as compared to compulsive masturbators (Bancroft and Vukadinovic, 2004).

Although the contributive role of non-sexual inhibitory mechanisms, such as harm aversion, has also been studied in the frame of compulsive sexual behavior (Miner et al., 2016; Rettenberger et al., 2016), the role of self-control has been neglected. This seems striking given that the lack of control lies at the core of the definition of compulsive sexual behavior. Self-control is the ability to control inner urges, immediate rewards and impulses to prioritize higher-order goals such as social harmony and long-term achievements. This capacity has been generally associated with better mental health, better interpersonal skills, better relationships and higher academic achievement (Tangney et al., 2004). Previous evidence suggests a potential association between compulsive sexual behavior and self-control. For instance, the comorbidity between hypersexuality and substance abuse is frequent (Kühn and Gallinat, 2016). Moreover, healthy individuals low in self-control report being more prone to fail at restraining their sexual behavior and also show a higher likelihood to engage in sexual infidelity (Gailliot and Baumeister, 2007). Although earlier studies have shown that high self-control is associated with less proneness to binge eating and alcohol abuse (Tangney et al., 2004), its association with compulsive sexual behavior proneness has been scarcely studied. To the best of our knowledge only one study has targeted this association, finding that higher levels of self-control were associated to more progress (number of “clean” months) in a 12-step therapy for individuals with compulsive sexual behavior (Efrati and Gola, 2018).

Among the biological factors contributing to compulsive sexual behavior proneness, the role of sexual hormones may be of particular relevance. Androgens affect sexuality both at a peripheral and central level. It has been shown that testosterone depletion in men not only alters levels of sexual functioning, but also decreases sexual cognition, sexual motivation, and sexual behavior (Schmidt et al., 2009; Jordan et al., 2011; Finkelstein et al., 2013). Similarly, hypogonodal men who are treated with testosterone show a higher sexual interest than non-treated patients (Redouté et al., 2005; Osterberg et al., 2014). Regarding the relationship between endogenous levels of testosterone and sexual motivation and behavior in healthy men, a large number of studies have revealed changes in testosterone levels in anticipation and as a consequence of sexual activity. For instance, testosterone levels in men increase after brief encounters with a woman (Roney et al., 2003), correlate with the viewing time of erotic stimuli (Rupp and Wallen, 2007), and increase during *in vivo* sexual stimulation and as consequence of sexual intercourse (Escasa et al., 2011). Note that null-findings have been reported as well (e.g., Goldey and van Anders, 2015), which could be explained by the relevance of the context, as it has been proposed that increased testosterone levels are manifested in sexual behavior mainly when there is competence or challenge involved (Wingfield et al., 1990; Goldey and van Anders, 2015). In spite of the extensive literature on the relationship between testosterone and sexual motivation, the literature addressing the association between compulsive sexual behavior and testosterone levels is surprisingly scarce. To our knowledge, only Chatzittofis et al. (2020) have addressed this point finding no differences in testosterone plasma levels between individuals diagnosed with hypersexual disorder and healthy controls, but they found that testosterone levels were significantly correlated with sexual compulsivity scores in hypersexual individuals.

In this study we aim to identify individual features that predict compulsive sexual behavior proneness in healthy young males from a biopsychological perspective. Whereas sexual excitation and inhibition have been extensively studied in the frame of compulsive sexual behavior, the contribution of self-control and testosterone have been largely ignored in spite of evidence pointing to a potential association and relevance. Therefore, we aim to investigate the simultaneous value of sexual excitation, sexual inhibition, self-control, and endogenous testosterone levels in the manifestation of compulsive sexual behavior as measured by the Sexual Compulsivity Scale. We hypothesize that whereas high sexual excitation and high testosterone levels can be associated with compulsive sexual behavior proneness, a high sexual inhibition and a high self-control may counteract and reduce such susceptibility. In addition, we aimed to explore the contribution of sexual excitation, sexual inhibition, self-control and testosterone, in predicting the frequency of solitary and non-solitary sexual behavior (i.e., masturbation and intercourse), as potential and distinctive manifestations of compulsive sexual behavior.

Since women and men significantly differ in their sexual cognition and inhibitory processes (Dewitte, 2016; Sjoberg and Cole, 2018) and women show less proneness to develop sexual compulsivity (Kuzma and Black, 2008), we only included men in our sample to provide a first test of the factors underlying sexual compulsivity proneness.

## Materials and Methods

### Participants

70 self-declared heterosexual male participants (18–35 years old, mean age = 24.7, *SD* = 4.4) were recruited through advertising in Maastricht University halls and in a Facebook group created to recruit participants for research at Maastricht University. Participants received ten euros in vouchers for their participation. The study was approved by the Ethics Review Committee Psychology and Neuroscience at Maastricht University and conformed to the Code of Ethics of the World Medical Association (Declaration of Helsinki). One participant was excluded due to an incomplete dataset. 92.1% of participants were Bachelor or Master students; of the remaining participants, 3 had completed university and 2 had completed high school. Approximately half of the participants were single (47.6%), the others had been in a relationship for an average period of 2.7 years (*SD* = 2.6).

### Procedure

Participants were instructed to abstain from eating, drinking any beverage (except water), brushing their teeth, and vigorously exercising 45 min before arrival and were suggested to drink water 10 min before to facilitate saliva collection. Appointments for the sessions were made at 9 or 10 a.m. to avoid variations due to the testosterone daily cycle.

At arrival, participants gave their written informed consent. Next, they were instructed to drool their saliva to the top of a 3.6 mL hormonal tube assay with the aid of a small straw. Following the saliva collection, participants performed computer-based tasks for a different study. After completing the tasks, participants were asked to fill in questionnaires on the computer. Participants were identified with one single four digits number as their ID and left alone while filling in the questionnaires to increase their comfort and confidence over the anonymity of their answers. After the experiment, saliva samples were stored at 4°C.

### Testosterone Assay

Centrifugation was performed at 2,000 g for 5 min and 250 μL supernatant from the sample was stored at −80°C until further LC–MS/MS analysis. For this later one, inter-assay coefficient of variation was 8.2% at 0.23 ng/dL (8 pmol/L) with a limit of quantification of 0.07 ng/dL (2.4 pmol/L).

### Questionnaires

#### Sexual Compulsivity Scale

This scale contains 10 items that measure the failure to control sexual impulses and interference in quotidian life because of sexual behavior (Example item: “My desires to have sex have disrupted my daily life”) (Kalichman and Rompa, 1995). These items are scored on a four-point Likert scale from Not at all like me to Very much like me. This scale has proven to have high reliability and be associated with sexual risky behaviors (Ballester-Arnal et al., 2013) (Cronbach’s alpha = 0.81).

#### Sexual Inhibition/Sexual Excitation Scales

This scale measures the individual propensity to be sexually aroused or sexually inhibited (Janssen et al., 2002). It contains one factor quantifying sexual excitation (20 items; Example item: “When an attractive person flirts with me, I easily become sexually aroused”) and two factors quantifying sexual inhibition: (1) SIS1—Inhibition derived from threat of sexual performance failure, distraction, or lack of physical stimulation (14 items; Example item: “Once I have an erection, I want to start intercourse right away before I lose my erection), and (2) SIS2—Inhibition due to the threat of performance consequences, such as risk of being caught, unwanted pregnancy, sexually transmitted diseases, feeling or causing pain, and partner’s too young age (11 items; Example item: “If there is a risk of unwanted pregnancy, I am unlike to get sexually aroused”). Answers were registered on a four-point Likert scale (ranging from 1 = strongly agree to 4 = strongly disagree). The raw scores were inversed so that higher scores indicate higher sexual excitation (SES, Possible range: 20–80) or inhibition (SIS1, Possible range: 14–56; SIS2, Possible range: 11–44) (SES—Cronbach’s alpha = 0.82; SIS2—Cronbach’s alpha = 0.73).

#### Brief Self-Control Scale

This scale consists of 13 items assessing the extent to which an individual is able to regulate his/her own behavior by resisting or inhibiting a preponderant response or desire in order to achieve long-term goals (Example item: “I do things that feel good in the moment but regret later on”). Participants answered in a five-point Likert scale ranging from Not at all to Very much. BSCS scores range from 13 to 65 with a higher number indicating more self-control. High internal consistency and test-retest reliability have been demonstrated for this scale (Tangney et al., 2004) (Cronbach’s alpha = 0.79).

#### Sexual Behavior and Self-Control Self-Reports

Participants reported the frequency of their solitary and dyadic sexual behavior by answering how often they: (a) Had masturbated, and (b) Had sexual intercourse, during the last 4 weeks through a five-point Likert scale ranging from Not once to Several times a day. As Supplementary Information of self-control, individuals reported in a 10- points scale to what extent they were able to control: (a) Their eating behavior, (b) Their monetary expenses, (c) Alcohol consumption, and (d) Drugs consumption.

## Results

### Descriptive Statistics

Table 1 displays the average, standard deviations and range of the self-report scores and testosterone levels. Whereas the levels of sexual inhibition (SIS1/2) in the current sample are similar to the reported in previous studies, the levels of sexual excitation are lower in our sample (see Janssen et al., 2002; Carpenter et al., 2008). Testosterone levels from the participants in this study were located within the expected range (present sample range:0.0316–0.7101 nmol/L; see González-Sánchez et al., 2015) and self-control scores were also similar to the reported in previous studies (Tangney et al., 2004; Fung et al., 2020). According to cutoff points (Kalichman and Rompa, 1995), 82% of our sample showed a score indicating no sexual compulsivity (score under 18 points), 12% had a score indicating mild sexual compulsivity (range: 18–23 points), 6% had a score indicating moderate sexual compulsivity (Range: 24–29 point) and no participant had a score that would indicate high levels of sexual compulsivity (higher than 30).

**TABLE 1:** Descriptive and comparative statistics of coupled and single men.

	**Total *n* = 69 mean (SD)**	**Total *n* = 69 range**	**Partnered men *n* = 35 mean (SD)**	**Single men *n* = 34 mean (SD)**	**T^p^**
Age	24.77 (4.41)	18–35	25.83 (4.61)	23.68 (3.93)	−2.07*
Testosterone (ng/dL)	7.57 (3.88)	0.91–20.48	6.7 (4.39)	8.42 (3.17)	1.58
SES	50.55 (7.32)	28–64	50.77 (5.96)	50.32 (8.58)	0.35
SIS1	27.35 (5.88)	28–54	26.63 (6.29)	28.09 (5.41)	−1.03
SIS2	29.74 (4.91)	19–43	29.03 (5.01)	30.47 (4.75)	−1.41
BSCS	41.57 (7.82)	25–61	40.97 (8.86)	42.18 (6.65)	0.21
Sexual compulsivity scale	14.74 (4.18)	10–27	15.06 (4.58)	14.41 (3.74)	−0.62
Masturbation frequency	3.71 (1.04)	1–6	3.63 (1.21)	3.79 (0.85)	0.37
Intercourse frequency	2.56 (1.49)	1–5	3.43 (1.17)	1.67 (1.24)	−6.01**

*SES, Sexual Excitation Scale raw scores; SIS1/2, Sexual Inhibition Scale Factors 1 and 2 raw scores; BSCS, Brief Self-Control Scale; Masturbation/Intercourse frequencies: 1- Not once, 2-Once or twice per month, 3-Once a week, 4-A few times a week, 5- Once a day, 6- Several times a day; ns, non-significant.*

*p = 0.04, **p = 0.001.

We explored whether single and partnered men differed in the psychological traits, testosterone levels, frequency of sexual behaviors, or sexual compulsivity scores with a series of independent *t*-tests. Single and partnered men differed only in the frequency of intercourse, with partnered men having significantly more sex than single men (*t* = −6.01, *p* = 0.001; Table 1).

### Correlation Analyses

**FIGURE 1:**
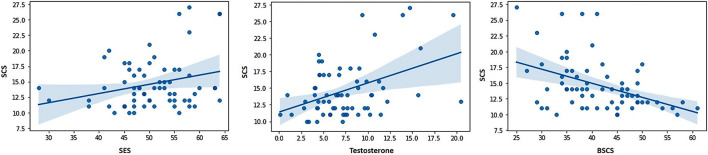
Scores from the Sexual Compulsivity Scale (SCS) were positively related to scores in the Sexual Excitation Scale (SES) and Testosterone, and negatively related to scores in the Brief Self-Control Scale (BSCS).

**FIGURE 2:**
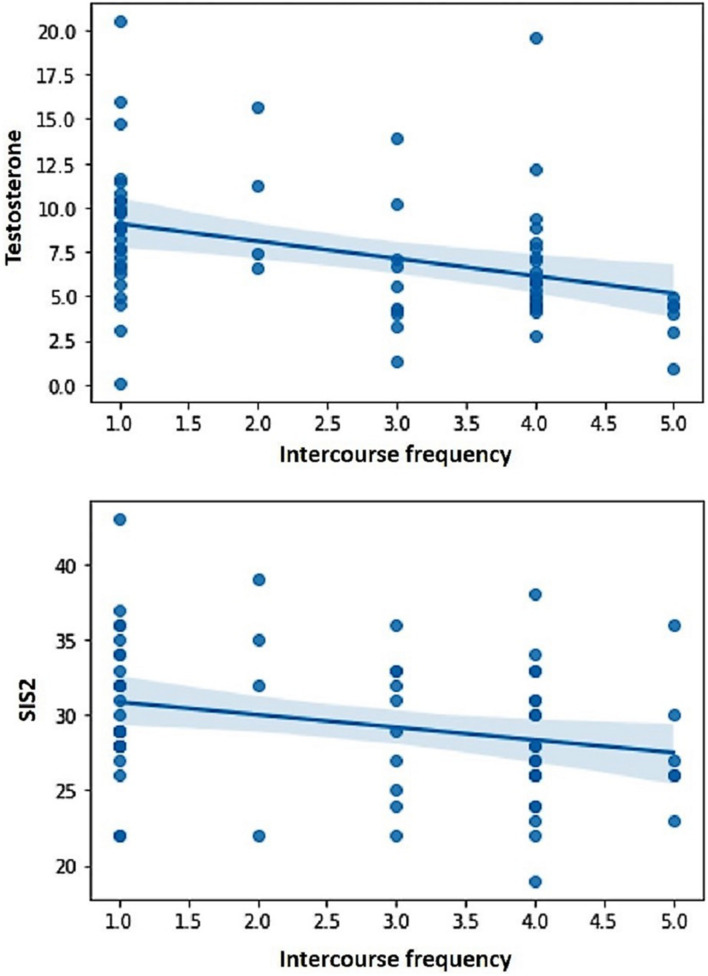
Intercourse frequency during the last month (in a scale from Not once to Several times a day) was negatively related to testosterone levels and scores in the Sexual Inhibition Scale, second factor (SIS2).

The correlations between the predictor variables (i.e., sexual excitation, sexual inhibition, self-control, and testosterone levels) and the dependent variables (i.e., sexual compulsivity scores, masturbation and intercourse frequencies) are displayed in Table 2 for the full sample and for single and partnered men separately. In the full sample, sexual compulsivity scores correlated positively with sexual excitation scores and with testosterone levels and correlated negatively with self-control scores (Figure 1). The two latter patterns were also observed in single and partnered men separately. In addition, intercourse frequency negatively related to the second factor of sexual inhibition and to testosterone (Figure 2). The inverse relationship between intercourse frequency and testosterone levels was also observed in partnered men but not in single men (SIS2).

**TABLE 2:** Correlations between predictor and dependent variables.

	**SES**	**SIS1**	**SIS2**	**BSCS**	**Test**
**Full sample**					
SCS	**0.26 (0.03)**	−0.027 (0.82)	0.01 (0.93)	−**0.47 (0.001)**	**0.34 (0.001)**
Masturbation	0.03 (0.76)	−0.07 (0.53)	−0.12 (0.31)	−0.03 (0.77)	0.13 (0.29)
Intercourse	0.001 (0.99)	−0.11 (0.41)	−**0.24 (0.03)**	−0.04 (0.73)	−**0.48 (0.001)**
**Single**					
SCS	0.27 (0.12)	0.23 (0.19)	−0.15 (0.38)	−**0.39 (0.02)**	**0.51 (0.004)**
Masturbation	0.15 (0.37)	−0.12 (0.49)	−0.26 (0.13)	−0.12 (0.48)	0.16 (0.38)
Intercourse	0.16 (0.36)	−0.21 (0.25)	−0.19 (0.27)	−0.03 0.86	−0.19 (0.31)
**Partnered**					
SCS	0.29 (0.09)	−0.07 (0.68)	0.13 (0.46)	−**0.51 (0.002)**	**0.41 (0.01)**
Masturbation	−0.05 (0.75)	−0.07 (0.69)	−0.07 (0.68)	0.01 (0.97)	0.12 (0.51)
Intercourse	−0.17 (0.33)	0.12 (0.49)	−0.28 (0.11)	0.06 (0.71)	−**0.36 (0.03)**

*Predictor variables: SES, Sexual Excitation Scale; SIS1/2, Sexual Inhibition Scale Factors 1 and 2; BSCS, Brief Self-Control Scale. Test., Testosterone. Dependent variables: SCS, Sexual Compulsivity Scale. For SCS, Pearson coefficients are reported and for Masturbation and Intercourse frequencies, Spearman coefficients are presented.*

*P-values are displayed in parentheses. Significant correlations (p ≤ 0.05) are highlighted in bold.*

We conducted complementary correlations between the sexual compulsivity scores and self-control questions targeting the ability of participants to control their eating, monetary expenses, and alcohol and drugs consumption (Supplementary Material). We also ran exploratory correlations between these variables and masturbation and intercourse frequencies and testosterone (Supplementary Table 1).

Finally, we conducted analyses to examine the relationship among the dependent variables (i.e., sexual compulsivity scores, masturbation and intercourse frequencies). There were no significant correlations or correlations close to significance (Table 3).

**TABLE 3:** Correlation between the independent variables.

	**Masturbation**	**Intercourse**
**Full sample**		
SCS	0.04 (0.77)	0.05 (0.68)
Masturbation	–	−0.03 (0.82)
**Single**		
SCS	0.21 (0.19)	0.08 (0.62)
Masturbation	–	−0.06 (0.68)
**Partnered**		
SCS	−0.05 (0.73)	0.05 (0.74)
Masturbation	–	−0.13 (0.39)

*SCS, Sexual Compulsivity Scale scores; Masturbation/Intercourse, Reported frequency of masturbation and intercourse frequencies. Values are Spearman coefficients with p-values displayed in parentheses.*

### Regression Analyses

Sexual excitation, sexual inhibition, self-control, and testosterone levels were simultaneously entered in three different stepwise regression models to predict compulsive sexual behavior proneness, and frequency of masturbation and intercourse. Due to sample size, regression analyses were only conducted in the full sample.

**FIGURE 3:**
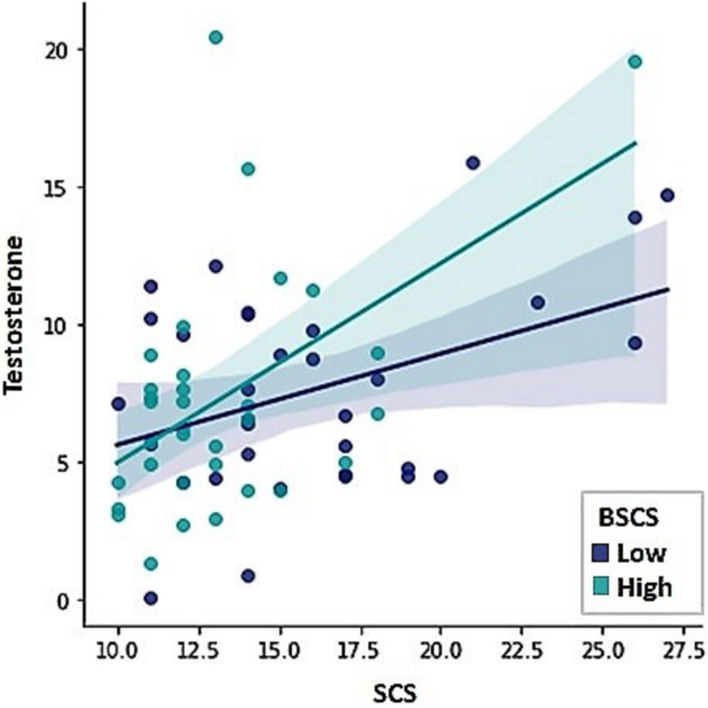
The Sexual Compulsivity Scale scores (SCS) were predicted by testosterone levels and scores in the Brief Self-Control Scale (BSCS).

Testosterone showed to be a positive predictor of sexual compulsivity proneness. The opposite pattern was found for self-control, indicating that higher scores on self-control were associated with lower sexual compulsivity scores (Table 4; *F* = 13.71, *R*^2^ = 0.29, *p* = 0.001). Because sexual excitation was significantly correlated with testosterone (*r* = 0.28, *p* = 0.02) and with sexual compulsivity scores (*r* = 0.26, *p* = 0.03), we conducted a Sobel test, to examine whether the relationship between testosterone and sexual compulsivity tendencies could be mediated by sexual excitation, which was not the case (indirect effect = 0.06, LI = 0.003, UI = 0.17). Figure 3 displays the relationship between testosterone and sexual compulsivity scores by low vs. high self-control (median split).

**TABLE 4:** Regression models for sexual compulsivity proneness reported levels, and masturbation and intercourse frequencies.

	**β**	**T**	***P***
**SCS**			
BSCS^a^	−0.42	−3.95	0.001
Testosterone ^a^	0.27	2.94	0.004
SES	−0.14	1.29	0.19
SIS2	−0.07	0.74	0.46
**Masturbation**			
BSCS	−0.06	−0.48	0.63
Testosterone	0.15	1.14	0.25
SES	−0.05	−0.42	0.67
SIS2	−0.16	−1.27	0.21
**Intercourse**			
Testosterone^a^	−0.36	−3.21	0.002
SIS2^a^	−0.26	−2.91	0.02
BSCS	−0.03	−0.46	0.64
SES	0.05	0.32	0.75

*SCS, Sexual Compulsivity Scale; BSCS, Brief Self-Control Scale; SES, Sexual Excitation Scale; SIS2, Sexual Inhibition Scale Factor 2.*

*^a^Resulting predictors from Stepwise Regressions.*

**FIGURE 4:**
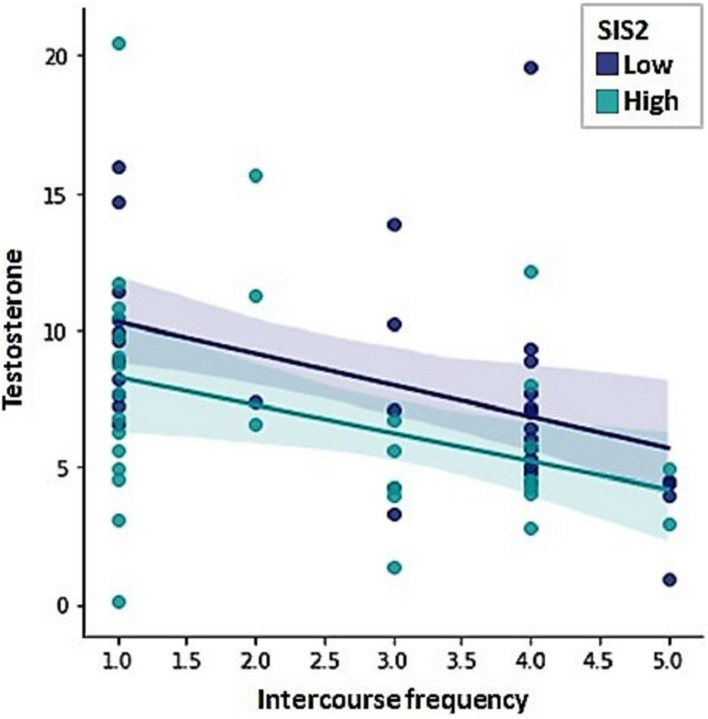
Intercourse frequency was negatively predicted by testosterone levels and Sexual Inhibition Scale, second factor (SIS2) scores.

None of the independent variables revealed a significant relation with frequency of masturbation. Intercourse frequency, on the other hand, was negatively predicted by testosterone levels and the sexual inhibition scores, indicating that the lower the level of sexual inhibition and the lower the level of testosterone levels, the more frequent intercourse men reported (Table 4; *F* = 10.26, *R*^2^ = 0.23, *p* = 0.001). Figure 4 displays the relationship between testosterone and intercourse frequency by low vs. high sexual inhibition (median split).

## Discussion

The aim of this study was to investigate possible determinants of sexual compulsivity proneness in young males. Drawing on a biopsychological perspective, we assessed the contribution of sexual excitation, sexual inhibition, self-control and testosterone. Our results showed that lower levels of self-control and higher levels of testosterone were associated with a higher compulsive sexual behavior proneness. Furthermore, whereas neither sexual excitation, sexual inhibition, self-control, nor testosterone predicted the frequency of masturbation, higher levels of sexual inhibition and testosterone levels did predict a lower frequency of sexual intercourse.

### Compulsive Sexual Behavior Proneness

Regarding the role of inhibitory mechanisms in predicting sexual compulsivity proneness, only lower levels of self-control predicted a higher tendency toward compulsive sexual behavior. This corresponds with the idea that self-control comprises the ability to control immediate reward in the pursue of long-term benefits and this ability predicts better adjustment, less addictive behaviors, and better personal relationships (Tangney et al., 2004). Moreover, this finding is in line with previous evidence regarding a relationship between trait self-control and sexual-restraint (understood as the control over undesirable and interfering sexual behavior; Gailliot and Baumeister, 2007) and a relationship between better self-control and advancement in therapy for compulsive sexual behavior (Efrati and Gola, 2018). Self-control targets a general ability to control temptations and behavior. Accordingly, the observed association with sexual compulsivity proneness might relate to the frequent comorbidity of substance abuse disorders in hypersexual individuals (Kühn and Gallinat, 2016) and to the fact that healthy individuals who report having poor self-control in non-sexual domains (e.g., meeting deadlines) also report being more likely to fail at controlling their sexual behavior (Gailliot and Baumeister, 2007). Our supplementary results did not show a relationship between sexual compulsivity proneness and the ability to control alcohol or drugs consumption, which may be due to the fact that we studied a non-clinical group of men. Nonetheless, we observed an association between sexual compulsivity proneness with the ability to control eating behavior, supporting the existence of a core self-control mechanism.

Sexual compulsivity proneness was also predicted by high testosterone levels. This finding is in partial agreement with Chatzittofis et al. (2020) who found a positive correlation between testosterone levels and sexual compulsivity scores in diagnosed hypersexual individuals but not in healthy individuals, which might be due to a disparity in their samples size (patients = 67 and healthy individuals = 39). Interestingly, the association between sexual compulsivity proneness and testosterone was not explained by a common association with sexual excitation. This may suggest that the association between sexual compulsivity and testosterone goes beyond a sexual context and might rather be explained by other factors. These factors may include sensation seeking, substance abuse and risk taking, as they have been associated with testosterone (Sakaguchi et al., 2006; Mehta et al., 2015; Tajima-Pozo et al., 2015; Kurath and Mata, 2018), and are often present in individuals with sexual compulsivity (Kafka, 2010). Furthermore, testosterone has been positively related to reinforcement sensitivity. Neuroimaging evidence supports a testosterone effect over the reward dopaminergic circuit during non-sexual motivational processing (Hermans et al., 2010). Thus, in some individuals, high testosterone may increase a susceptibility to seek and, under certain contexts, abuse rewarding stimuli. Our supplementary findings shown that high testosterone was related to a low ability to control alcohol (single men) and drugs consumption (full sample). However, these are incidental findings and therefore should be interpreted with caution.

Contrary to our expectations, sexual compulsivity proneness was not predicted by sexual excitation and sexual inhibition. Although sexual excitation showed a mild correlation with sexual compulsivity proneness, it was not a significant predictor in the regression model, which may be due to its common correlation with testosterone. Another factor may have been the relatively low sexual excitation levels in the current sample (as compared to previous ones: Janssen et al., 2002; Carpenter et al., 2008), potentially due to cultural differences. The lack of association between sexual compulsivity proneness and sexual inhibition was less surprising as null findings or weak correlations with sexual compulsivity or hypersexuality are not uncommon (Bancroft and Vukadinovic, 2004; Pachankis et al., 2014; Miner et al., 2016; Rettenberger et al., 2016; Efrati and Mikulincer, 2018). This common finding is important as it points to a core distinction between these two domains: whereas sexual inhibition (second factor) mostly tackles the regulation of sexual arousal under specific risky circumstances and predominantly during sexual encounters, compulsive sexual behavior refers to the lack of regular control of non-specified sexual behavior (solitary or dyadic) and its constant interference with daily life goals. Although both domains may be simultaneously present in some individuals, that does not seem to be the norm.

### Masturbation and Intercourse

We also explored the contributing role of sexual excitation, sexual inhibition, self-control, and testosterone levels in predicting masturbation and intercourse frequencies. Surprisingly, none of the variables explained the frequency of masturbation. It may be that sexual excitation and sexual inhibition did not have predictive value because they exert their influence mainly in contexts involving other people (e.g., getting aroused by the touch of somebody or getting sexually inhibited during intercourse). Although being an easily accessible rewarding behavior, masturbation frequency did not relate to self-control. Moreover, testosterone levels did not predict the frequency of masturbation which supports that testosterone does not correspond with a mere physiological sexual drive. It is possible that the frequency of masturbation is related more to social attitudes and mood regulation than only biologically driven sexual arousability.

Intercourse frequency was negatively predicted by sexual inhibition (second factor) and by testosterone levels. Sexual inhibition tackles the regulation of sexual arousal mostly in interpersonal encounters and therefore individuals with low sexual inhibition are likely more prone to seek and maintain sexual encounters in spite of adverse consequences.

Contrary to what is commonly expected, testosterone showed a negative relation with intercourse frequency. This pattern -less testosterone in more sexually active men- has been reported previously (Kraemer et al., 1976; Sakaguchi et al., 2007; Puts et al., 2015). Other studies have revealed an interesting pattern of findings on this behalf. When exploring the association between sexual responding and testosterone within the same individuals, testosterone levels increased after sexual stimulation and orgasmic activity (Kraemer et al., 1976; Escasa et al., 2011). However, when considering the association between testosterone and sexual activity across different individuals, the opposite pattern is found, with testosterone levels being higher in less sexually active individuals (Kraemer et al., 1976). These observations may be explained in the frame of the challenge hypothesis, which states that testosterone variations in response to mating and reproductive behaviors are not absolute, but that they are related to those behaviors in the context of challenging environments, as they would prepare the organism for mating process challenges (Wingfield et al., 1990). Interestingly, this association was observed in partnered but not in single men, with individuals low in testosterone reporting a higher sexual intercourse frequency. According to the challenge hypothesis, this would indicate that partnered men with more frequent intercourse do not perceive a competing context and therefore their organisms do not prepare for challenges.

Finally, we investigated masturbation and intercourse frequencies, as sexual compulsivity can manifest in individual-based or partnered behaviors (Efrati and Mikulincer, 2018). However, masturbation and intercourse frequencies did not relate to compulsive sexual behavior proneness. This seems to highlight that it is the inadequacy of the context, the excessive preoccupation and the interference with daily life goals that are at the core of problematic sexual behavior rather than a high frequency of sexual behaviors *per se*.

In sum, this study showed that self-control and testosterone are associated with sexual compulsivity proneness, potentially as a protective and risk factor, respectively. Interestingly, whereas sexual excitation, sexual inhibition, and to a lesser extent testosterone, have been studied in clinical samples, this is not the case for self-control. Based on our results, future clinical studies may target this trait and assess whether entrainment of self-control might be a particularly useful tool in the prevention and treatment of compulsive sexual behavior. In addition, clinical studies may include testosterone monitoring and its possible association with diet and exercise, and investigate how this relates to sexual compulsive behavior symptomatology. As androgen deprivation therapy poses the risk of harmful side-effects, its consideration could be reserved to severe cases of diagnosed compulsive sexual behavior. On a more general level, the current study underscores the simultaneous influence of psychological traits and biological factors in modeling and shaping sexual behavior. This showcases the need for a biopsychological perspective by simultaneously studying psychological and biological factors in order to increase our understanding of the individual factors that contribute to a higher sexual compulsive proneness.

### Limitations

One limitation of this study is that we tested only heterosexual male participants. Although compulsive sexual behavior is more common in men than in women (Kuzma and Black, 2008), future studies can investigate whether the same factors (self-control and testosterone) predict compulsive sexual behavior in women and in individuals with different sexual orientations as well. A second limitation is the correlational nature of the present findings, so, although some possible explanations were discussed, we cannot make direct causal inferences. Third, we used salivary samples to assess testosterone which does not give a direct indicator of serum levels. However, salivary measurements derived from passive drooling have shown to be reasonably valid for behavioral studies in men (Fiers et al., 2014). Finally, our study comprises a non-clinical sample, future studies can assess whether our results extend to individuals diagnosed with compulsive sexual behavior.

## Data Availability Statement

The raw data supporting the conclusions of this article will be made available by the authors, without undue reservation.

## Ethics Statement

The studies involving human participants were reviewed and approved by the ECP 142-01-07-2014. The patients/participants provided their written informed consent to participate in this study.

## Author Contributions

GR-N, MD, AS, and TS contributed to the protocol design, results interpretation, and manuscript review. GR-N collected, analyzed the data, and drafted the manuscript. All authors contributed to the article and approved the submitted version.

## Conflict of Interest

The authors declare that the research was conducted in the absence of any commercial or financial relationships that could be construed as a potential conflict of interest.

## Publisher’s Note

All claims expressed in this article are solely those of the authors and do not necessarily represent those of their affiliated organizations, or those of the publisher, the editors and the reviewers. Any product that may be evaluated in this article, or claim that may be made by its manufacturer, is not guaranteed or endorsed by the publisher.
