# Supplementary material for: Individual Differences in Testosterone and Self-Control Predict Compulsive Sexual Behavior Proneness in Young Males
Source: Front Psychol. 2021 Dec 3;12:723449. doi: 10.3389/fpsyg.2021.723449 (PMC8677662; doi:10.3389/fpsyg.2021.723449)
Supplement: Supplementary file 1 [file Table_1.DOCX]

Supplementary Material

**Supplementary Table 1.** Pearson correlations between supplementary self-control variables and dependent variables.

|  | | **Eating** | **Money** | **Alcohol** | **Drugs** |
| --- | --- | --- | --- | --- | --- |
| ***Full Sample*** |  | |  |  |  |
| **SCS** | **-.31**  **.01** | | -.15  (.21) | -.19  (.12) | -.21  (.08) |
| **BSCS** | **.45**  **(.001)** | | **.42**  **(.001)** | **.33**  **(.005)** | **.38**  **(.001)** |
| **Masturbation** | -.17  (.15) | | -.05  (.65) | -.09  .42 | -.09  (.43) |
| **Intercourse** | .05  (.65) | | -.02  (.82) | .12  (.32) | .09  (.43) |
| **Testosterone** | -.15  (.20) | | -.05  .65 | -.19  (.12) | **-.29**  **(.01)** |
| ***Single*** |  | |  |  |  |
| **SCS** | **-.41**  **(.01)** | | -.22  (.20) | -.14  (.40) | **-.37**  **(.03)** |
| **BSCS** | **.62**  **(.001)** | | **.47**  **.004** | .24  (.16) | **.34**  **(.05)** |
| **Masturbation** | -.09  (.61) | | .07  (.67) | -.09  (.58) | -.08  (.62) |
| **Intercourse** | .09  (.59) | | .01  (.95) | .14  (.41) | .07  (.69) |
| **Testosterone** | -.31  (.09) | | -.24  (.19) | **-.37**  **(.04)** | **-.46**  **(.01)** |
| ***Partnered*** |  | |  |  |  |
| **SCS** | -.23  (.17) | | -.08  (.61) | -.24  (.15) | -.09  (.57) |
| **BSCS** | **.36**  **(.03)** | | **.37**  **(.02)** | **.43**  **.01** | **.45**  **.007** |
| **Masturbation** | -.22  (.19) | | -.15  (.38) | -.09  .58 | -.10  (.55) |
| **Intercourse** | .11  (.52) | | .04  (.78) | .04  (.78) | .10  (.56) |
| **Testosterone** | -.10  (.56) | | .03  (.82) | -.04  (.79) | -.16  (.35) |

Participants reported their own perceived ability to control their eating behaviour, monetary expenses, and alcohol and drugs consumption. SCS – Sexual Compulsivity Scale. BSCS – Brief Self-Control Scale. P-values are displayed in parenthesis.
